# Laryngeal Cancer in Europe and Its Subregions: A Temporal Epidemiological Analysis Based on Global Burden of Disease Data

**DOI:** 10.7759/cureus.92077

**Published:** 2025-09-11

**Authors:** Christos S Avdulla, Nicholas Mastronikolis, Ntaniela Tachirai, Michalis Leotsinidis, Eleni Jelastopulu

**Affiliations:** 1 Department of Public Health, University of Patras, Patras, GRC; 2 Department of Otorhinolaryngology, University of Patras, Patras, GRC; 3 Infectious Diseases Unit, University General Hospital "Attikon", Athens, GRC

**Keywords:** epidemiology, incidence, laryngeal cancer, mortality, mortality-to-incidence ratio

## Abstract

Introduction

Laryngeal cancer remains a significant public health issue in Europe, with considerable variation across countries and regions. Evaluating trends in incidence, mortality, and survival indicators such as the mortality-to-incidence ratio (MIR) may provide insights into disparities and guide policy development. The objective of this study was to conduct a 25-year epidemiological data analysis of age-standardized rates (ASRs) for the incidence and mortality of laryngeal cancer, and to compare the MIRs across European countries and subregions (North, South, East, and West Europe), using data from the Global Burden of Disease (GBD) database.

Methods

We extracted secondary data on laryngeal cancer from the GBD dataset provided by the Institute for Health Metrics and Evaluation (IHME) for the years 1997 to 2021. ASRs for incidence and mortality, as well as MIRs based on these ASRs, were analyzed. Descriptive statistics (mean, median, standard deviation, minimum-maximum range) and linear regression were performed using Microsoft Excel 365.

Results

All key indicators showed a downward trend over the 25-year period. The average MIR declined from 0.563 in 1997 to 0.459 in 2021 (R² = 96.26%, p < 0.001). Northern Europe improved from 0.448 to 0.355, while Western Europe consistently reported the lowest MIRs (0.388 to 0.326), likely reflecting improved access to specialized oncology services, advanced treatment protocols, and more equitable healthcare delivery. Finland (0.252), France (0.263), Ireland (0.274), Sweden (0.276), and Norway (0.278) had the most favorable MIRs. Eastern Europe, despite improvement (from 0.690 to 0.565), still exhibited the highest MIRs. Southern Europe also showed a decrease (0.575 to 0.465), although countries like Montenegro and Serbia reported the highest incidence rates (up to 9.9 cases per 100,000).

Conclusion

Substantial geographical disparities were observed in laryngeal cancer incidence, mortality, and management across Europe. Northern countries demonstrate better preventive strategies, while Western Europe shows more effective disease management. These findings underscore the need for targeted public health interventions focusing on prevention, early diagnosis, reducing inequalities, and improving access to quality cancer care.

## Introduction

Laryngeal cancer is among the most common malignancies of the head and neck and represents a major cancer of the upper aerodigestive tract [1-2]. Epidemiological studies over time have associated its development with multiple behavioral and environmental risk factors, including long-term tobacco and alcohol use, exposure to air pollutants, particularly occupational hazards, and broader environmental and dietary influences. Additionally, genetic predisposition and certain viral infections, particularly high-risk human papillomavirus (HPV) and, to a lesser extent, Epstein-Barr virus (EBV), have been recognized as important contributors to the pathogenesis of laryngeal cancer [3-4]. The multifactorial nature of carcinogenesis is particularly important, as the co-occurrence of multiple risk factors significantly increases the likelihood of developing laryngeal cancer [1,3-4].

Despite advances in early detection and treatment, laryngeal cancer continues to impose a considerable global epidemiological burden. According to recent estimates from GLOBOCAN and the World Cancer Research Fund (WCRF), laryngeal cancer ranks among the 20 most common cancers worldwide. In 2022, a total of 189,191 new cases were reported globally, with an age-standardized incidence rate (ASIR) of 1.9 per 100,000 population. Among males, it ranks 16th, with 165,794 new cases (ASIR = 3.5), while among females, it ranks 25th, with 23,397 cases (ASIR = 0.45) [5-6].

Marked geographical variation is also observed, with Europe reporting the highest ASIR for laryngeal cancer at 2.7 per 100,000, followed by Latin America and the Caribbean (2.1), North America (1.9), Asia (1.8), and Africa (1.3) [5]. A similar pattern is seen in mortality, where Europe’s ASIR is 1.21 per 100,000, slightly lower than that of Latin America and the Caribbean (1.23), but higher than in Asia (1.02), Africa (0.9), and North America (0.56) [5].

Europe’s leading position in both incidence and mortality rates, along with substantial regional disparities, underscores the importance of robust population-level data analysis to support effective and targeted public health interventions at both national and regional levels. Within this context, the present study aims to analyze and compare key epidemiological indicators, incidence, mortality, and the mortality-to-incidence ratio (MIR), for laryngeal cancer across European countries and subregions.

## Materials and methods

Epidemiological data: incidence and mortality

Epidemiological data on the incidence and mortality of laryngeal cancer were obtained from the Global Burden of Disease (GBD) database, curated by the Institute for Health Metrics and Evaluation (IHME) [7]. The analysis covered the period from 1997 to the most recent available year, 2021, providing 25 years of data for both sexes.

To eliminate the effect of differences in age structure between countries and to ensure the comparability of data, the analysis employed age-standardized rates. Specifically, incidence was assessed using the age-standardized incidence rate (ASIR), and mortality was analyzed using the age-standardized mortality rate (ASMR).

Mortality-to-incidence ratio

The mortality-to-incidence ratio is an important epidemiological indicator used to assess the severity of a disease and the effectiveness of diagnostic and therapeutic interventions. It compares the number of deaths from a disease to the number of newly diagnosed cases within the same calendar year.

MIR can be calculated in two ways: unadjusted and age-adjusted [8-9]. In this study, the age-adjusted MIR was used, calculated as the ratio of the age-standardized mortality rate to the age-standardized incidence rate: MIR = ASMR/ASIR, where ASMR is the number of deaths per 100,000 population, adjusted for differences in age distribution, and ASIR is the number of new cases per 100,000 population, also adjusted for age distribution. The analysis of the MIR for laryngeal cancer was based on this formula, using data from the GBD database for the period 1997-2021 [7]. A high MIR indicates that a large proportion of patients diagnosed with this type of cancer die from the disease. Conversely, a lower MIR reflects improved survival and better disease management, as the number of deaths is lower relative to new diagnoses [8-9].

In our study, a “high MIR” was defined as a value exceeding the overall European mean MIR for the corresponding year, indicating a higher proportion of mortality relative to incidence.

European countries included in the study

All European countries included in the GBD database were analyzed, according to the classification defined by the Institute for Health Metrics and Evaluation [7], totaling 48 countries. Specifically, the countries analyzed were as follows: Albania, Andorra, Armenia, Austria, Azerbaijan, Belarus, Belgium, Bosnia and Herzegovina, Bulgaria, Croatia, Cyprus, Czechia, Denmark, Estonia, Finland, France, Georgia, Germany, Greece, Greenland, Hungary, Iceland, Ireland, Italy, Latvia, Lithuania, Luxembourg, Malta, Monaco, Montenegro, Netherlands, North Macedonia, Norway, Poland, Portugal, Republic of Moldova, Romania, Russian Federation, San Marino, Serbia, Slovakia, Slovenia, Spain, Sweden, Switzerland, Turkey, Ukraine, and the United Kingdom.

Regional classification of European countries

The countries included in the study were grouped into four major European subregions, Southern, Northern, Eastern, and Western Europe, based on the United Nations geoscheme [10], provided that data on laryngeal cancer were available in the GBD database. The classification was as follows:

Northern Europe (10 of 16 regions): Denmark, Estonia, Finland, Iceland, Ireland, Latvia, Lithuania, Norway, Sweden, and the United Kingdom. The following territories were excluded due to a lack of data: Åland Islands, Faroe Islands, Guernsey, Isle of Man, Jersey, and Svalbard and Jan Mayen Islands.

Southern Europe (14 of 16 countries): Albania, Andorra, Bosnia and Herzegovina, Croatia, Greece, Italy, Malta, Montenegro, North Macedonia, Portugal, San Marino, Serbia, Slovenia, and Spain. Gibraltar and the Holy See (Vatican City) were excluded due to unavailable data.

Eastern Europe (10 countries): Belarus, Bulgaria, Czechia, Hungary, Poland, Republic of Moldova, Romania, Russian Federation, Slovakia, and Ukraine.

Western Europe (8 of 9 countries): Austria, Belgium, France, Germany, Luxembourg, Monaco, Netherlands, and Switzerland. Liechtenstein was excluded due to a lack of data.

Statistical analysis

Statistical analysis was performed using Microsoft Office Excel 365 (Microsoft Corporation, Redmond, USA). Although Excel has limitations in advanced statistical modeling, it was deemed appropriate for the descriptive and regression analyses applied in this study, which do not require advanced statistical modeling. Descriptive statistics were used to summarize the data, including mean, median, standard deviation (SD), minimum and maximum values, and range. Linear regression analysis was also applied to assess trends over time, with underlying assumptions (linearity, independence, homoscedasticity, and normality of residuals) evaluated and deemed adequately satisfied. Statistical significance was determined using a p-value threshold of ≤0.05. Additionally, for each indicator (incidence, mortality, and MIR), the mean value for Europe as a whole was compared with the mean values of the four geographic subregions (Southern, Northern, Eastern, and Western Europe).

Ethical considerations

When using the data from the GBD database, maintained by the IHME, we adhered to the established guidelines for ethical data usage. The GBD database provides open access to epidemiological data for scientific research, under the condition that proper citation is given and terms of use are respected. Compliance with these principles ensures the transparency and credibility of the study, as well as alignment with IHME's standards for data protection and scientific integrity.

## Results

Incidence of laryngeal cancer in Europe

The age-standardized incidence rates of laryngeal cancer for both sexes across all European countries are presented in Table 1. During the study period (1997-2021), the highest mean ASIR was recorded in Monaco, at 11.49 cases per 100,000 population, with a low of 10.44 in 2020 and a peak of 13.61 in 1997. Montenegro ranked second with a mean ASIR of 9.31 (SD = 0.34), ranging from 8.71 in 2021 to 9.90 in 2003. Serbia followed in third place, with a mean of 6.19, a minimum of 5.55 in 2021, and a maximum of 6.72 in 2007.

**Table 1:** Age-standardized incidence of laryngeal cancer per 100,000 population in European countries (both sexes, 1997–2021), ranked from highest to lowest mean value ASIR: age-standardized incidence rate; SD: standard deviation ^a^Minimum and maximum values with corresponding years ^b^Country ranking based on average ASIR

Country	Mean ASIR	Median	SD	Minimum^a^	Year of Minimum	Maximum^a^	Year of Maximum	Rank^b^
Monaco	11.486	11.46	0.842	10.44	2020	13.61	1997	1
Montenegro	9.307	9.23	0.337	8.71	2021	9.9	2003	2
Serbia	6.191	6.19	0.347	5.55	2021	6.72	2007	3
Hungary	6.062	6.38	0.614	5.1	2016	6.99	1999	4
North Macedonia	5.987	6.05	0.410	5.0	2021	6.52	2006	5
Spain	5.698	5.6	1.110	4.13	2020	7.69	1997	6
France	5.673	5.51	0.646	4.82	2021	7.25	1998	7
Bulgaria	5.408	5.61	0.477	4.51	1999	6.07	2008	8
Romania	5.327	5.42	0.490	4.32	2000	5.89	2019	9
Croatia	5.257	5.04	0.768	4.09	2020	6.6	1997	10
Greece	5.146	5.26	0.421	4.47	2019	5.68	1999	11
Poland	4.810	4.76	0.581	3.92	2021	5.59	1999	12
Bosnia and Herzegovina	4.727	4.74	0.166	4.27	2021	5.09	2007	13
San Marino	4.593	4.55	0.764	2.64	2021	5.92	1997	14
Belarus	4.582	4.21	0.860	3.5	2015	6.46	1998	15
Georgia	4.281	4.24	0.325	3.36	2007	5.08	2016	16
Slovakia	4.162	4.15	0.457	3.52	2020	4.94	1998	17
Italy	3.990	4.03	0.707	2.97	2020	5.38	1997	18
Belgium	3.930	3.83	0.754	2.74	2020	5.64	1997	19
Lithuania	3.909	3.94	0.335	3.36	2017	4.51	2007	20
Republic of Moldova	3.784	3.82	0.219	3.35	2005	4.13	2012	21
Ukraine	3.744	3.52	0.674	2.88	2021	5.2	1997	22
Luxembourg	3.708	3.55	0.549	2.82	2020	4.92	1997	23
Slovenia	3.646	3.69	0.326	3.05	2014	4.21	1997	24
Estonia	3.636	3.62	0.344	3.15	2013	4.54	1998	25
Armenia	3.584	3.79	0.597	2.44	2021	4.43	1999	26
Turkey	3.579	3.49	0.291	3.3	2018	4.34	1997	27
Russian Federation	3.454	3.31	0.473	2.88	2021	4.46	1997	28
Latvia	3.373	3.37	0.323	2.8	2019	3.96	1998	29
Albania	3.334	3.33	0.125	3.11	2009	3.54	2017	30
Portugal	3.297	3.44	0.510	2.44	2021	4.1	1997	31
Czechia	3.113	3.11	0.332	2.46	2020	3.63	1997	32
Denmark	3.064	3.07	0.357	2.54	2021	3.85	1997	33
Andorra	2.929	2.83	0.352	2.16	2021	3.67	1997	34
United Kingdom	2.878	2.86	0.160	2.51	2020	3.16	1997	35
Azerbaijan	2.842	2.85	0.291	2.25	2021	3.42	1997	36
Netherlands	2.794	2.75	0.415	2.14	2020	3.54	1997	37
Malta	2.793	2.73	0.377	2.2	2020	3.43	1997	38
Ireland	2.716	2.74	0.201	2.28	2020	3.09	1997	39
Germany	2.680	2.67	0.313	2.23	2021	3.37	1997	40
Cyprus	2.455	2.44	0.136	2.14	2021	2.67	2009	41
Austria	2.446	2.33	0.350	1.93	2021	3.12	1997	42
Switzerland	2.429	2.38	0.350	1.77	2020	2.98	1997	43
Greenland	2.175	2.15	0.163	1.87	2021	2.59	1997	44
Norway	1.722	1.66	0.235	1.33	2021	2.1	1997	45
Finland	1.648	1.63	0.132	1.44	2013	1.96	1997	46
Iceland	1.498	1.47	0.212	1.25	2015	2.0	1997	47
Sweden	1.218	1.22	0.103	0.98	2021	1.4	2004	48

Hungary ranked fourth, with a mean ASIR of 6.06 (SD = 0.61), ranging from 5.10 in 2016 to 6.99 in 1999. North Macedonia followed closely with a mean of 5.99 (SD = 0.41), ranging from 5.00 in 2021 to 6.52 in 2006. Spain ranked sixth, with a mean ASIR of 5.70 (SD = 1.11), varying from 4.13 in 2020 to 7.69 in 1997. Several other countries, including France, Bulgaria, and Romania, also reported elevated average incidence rates. For example, France had a mean ASIR of 5.67, with a minimum of 4.82 in 2021 and a maximum of 7.25 in 1998. Greece ranked 11th overall, with a mean ASIR of 5.15, ranging from 4.47 in 2019 to 5.68 in 1999.

At the lower end of the spectrum were Northern European countries, such as Sweden (mean ASIR = 1.22) and Iceland (1.50). Finland and Norway also reported relatively low average rates, with means of 1.65 and 1.72, respectively.

**Figure 1:**
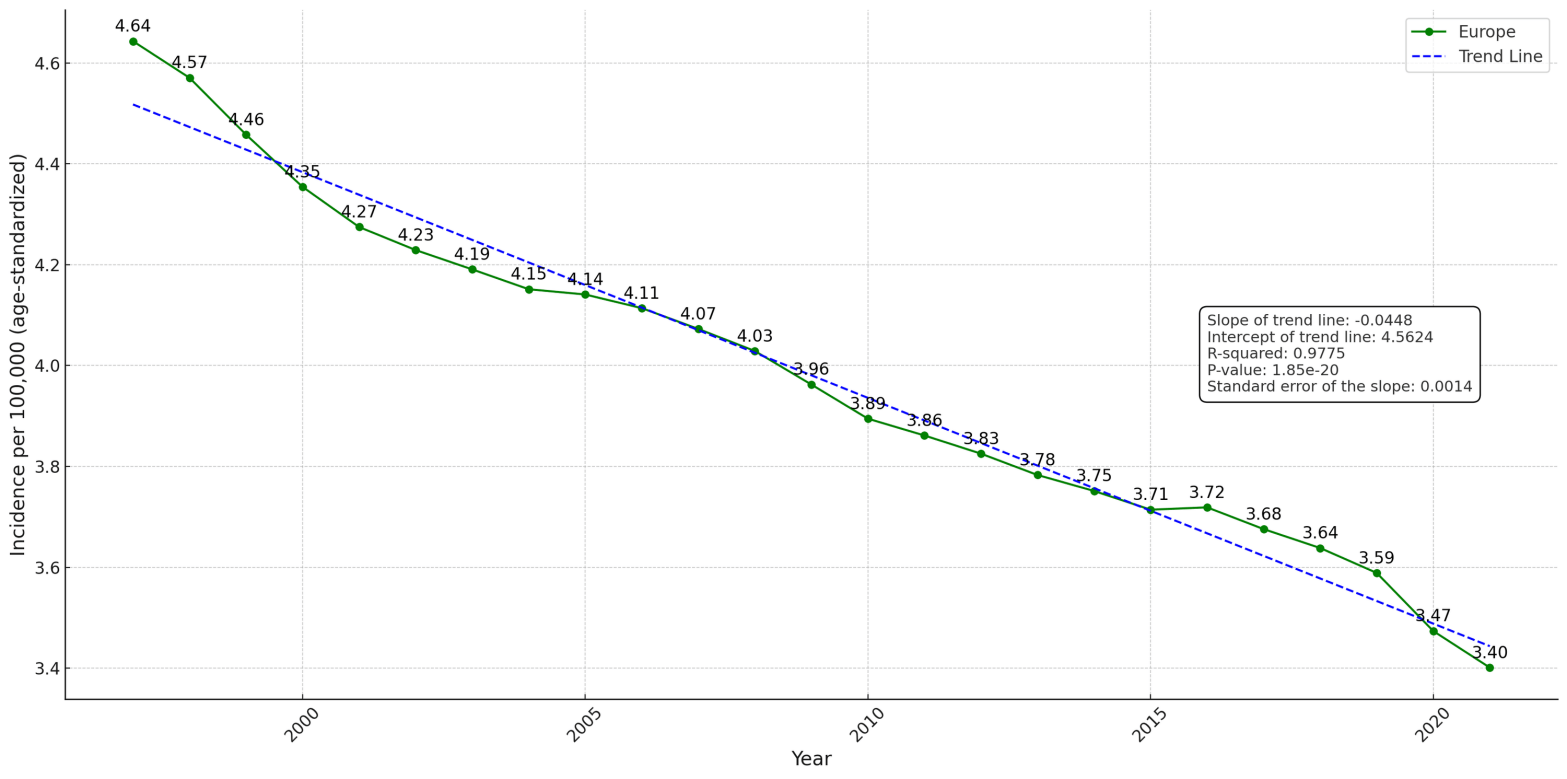
Trends in the mean age-standardized incidence of laryngeal cancer per 100,000 population in Europe (1997–2021) The dashed blue line indicates the linear trend; the green line shows the observed age-standardized incidence rate (ASIR).

A consistent downward trend in the ASIR of laryngeal cancer was observed across all European countries over the study period. Specifically, the mean incidence rate declined from 4.64 cases per 100,000 population in 1997 to 3.40 in 2021 (Figure 1). This downward trajectory was confirmed through linear regression analysis. The slope of the trend line was -0.0448, indicating an average annual decrease in incidence, with an intercept of 4.5624. The R² (R-squared) value of 0.9775 (97.75%) suggests an excellent fit of the data to the linear model. Furthermore, the p-value was <0.001, confirming that the decreasing trend is statistically significant. The standard error of the slope was 0.0014, further supporting the precision of the estimate.

**Figure 2:**
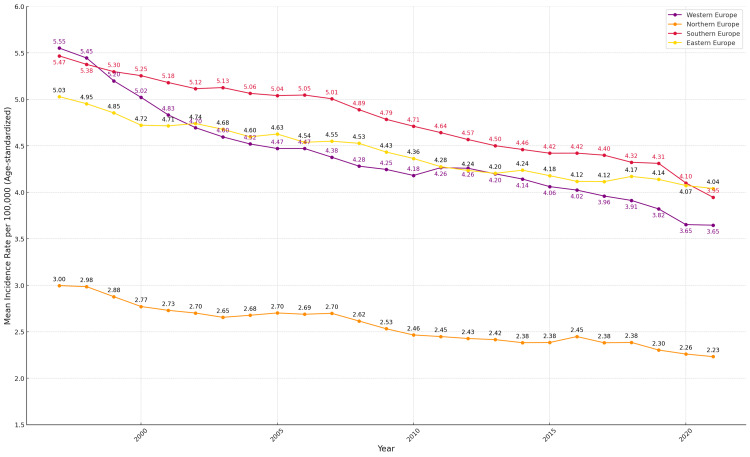
Age-standardized incidence of laryngeal cancer per 100,000 population across European subregions, according to United Nations classification, 1997–2021 Regions shown: Eastern Europe (yellow line), Northern Europe (orange line), Southern Europe (red line), and Western Europe (purple line)

Further analysis, as shown in Figure 2, presents trends in the age-standardized incidence of laryngeal cancer per 100,000 population across the four European subregions defined by the United Nations: Southern, Western, Northern, and Eastern Europe, from 1997 to 2021.

Southern and Western Europe began the study period with the highest average incidence rates, both exceeding 5 cases per 100,000 in 1997. Over time, both regions exhibited a steady decline. By 2021, the incidence in Western Europe had decreased to 3.65 cases per 100,000, while that in Southern Europe had declined to 3.95.

Northern Europe and Eastern Europe started with lower incidence rates-approximately 3 cases per 100,000 in Northern Europe and slightly above 5 in Eastern Europe in 1997. Northern Europe showed a steady decline throughout the study period, reaching 2.23 cases per 100,000 in 2021. Although Eastern Europe also demonstrated a downward trend, its incidence remained higher than that of Northern Europe, ending at around 4 cases per 100,000.

By the end of the observation period, incidence rates in Southern, Eastern, and Western Europe remained above the overall European average.

Mortality rates for laryngeal cancer in Europe

The analysis of laryngeal cancer mortality data across Europe offers a comparative overview among both European subregions and individual countries, aiming to highlight long-term trends and region-specific characteristics. Table 2 presents the age-standardized mortality rates for laryngeal cancer per 100,000 population across all European countries, for both sexes, over the study period. The highest average mortality rates were observed in Montenegro and North Macedonia. Specifically, Montenegro recorded the highest mean ASMR at 5.39 deaths per 100,000, ranging from 6.07 in 2003 to 4.67 in 2021. North Macedonia followed with a mean ASMR of 4.15, with values ranging from a low of 3.2 in 2021 to a peak of 4.8 in 1998.

**Table 2:** Age-standardized mortality rates (ASMR) for laryngeal cancer per 100,000 population across all European countries (both sexes, 1997–2021), ranked from the highest to lowest mean value ASMR: age-standardized mortality rate; SD: standard deviation ^a^Minimum and maximum values with corresponding years ^b^Country ranking based on average ASMR

Country	Mean ASMR	Median	SD	Minimum^a^	Year of Minimum	Maximum^a^	Year of Maximum	Rank^b^
Montenegro	5.394	5.36	0.433	4.67	2021	6.07	2003	1
North Macedonia	4.154	4.23	0.498	3.2	2021	4.8	1998	2
Monaco	3.852	3.78	0.458	3.28	2020	4.85	1997	3
Serbia	3.563	3.55	0.507	2.79	2021	4.43	1997	4
Hungary	3.400	3.47	0.504	2.71	2021	4.24	1999	5
Poland	3.260	3.21	0.532	2.5	2021	4.07	1997	6
Bulgaria	3.236	3.22	0.212	2.87	2012	3.69	2008	7
Georgia	3.230	3.24	0.232	2.57	2007	3.83	2016	8
Bosnia and Herzegovina	3.162	3.14	0.301	2.64	2021	3.9	1997	9
Romania	3.137	3.11	0.132	2.92	2000	3.56	1997	10
Croatia	2.940	2.77	0.633	2.06	2021	4.12	1997	11
Belarus	2.912	2.68	0.818	1.96	2015	4.56	1998	12
Republic of Moldova	2.716	2.72	0.190	2.44	2021	3.15	1997	13
Armenia	2.713	2.82	0.561	1.7	2021	3.54	1999	14
Slovakia	2.596	2.58	0.439	2.01	2020	3.34	1998	15
Lithuania	2.589	2.63	0.306	2.11	2021	3.11	1997	16
Ukraine	2.477	2.24	0.563	1.79	2021	3.6	1997	17
Azerbaijan	2.374	2.4	0.324	1.76	2021	2.93	1997	18
Russian Federation	2.368	2.27	0.548	1.67	2021	3.37	1997	19
Turkey	2.326	2.18	0.477	1.77	2021	3.41	1997	20
Albania	2.324	2.25	0.203	2.05	2021	2.73	1997	21
Portugal	2.300	2.33	0.496	1.57	2020	3.16	1997	22
Spain	2.250	2.11	0.620	1.49	2020	3.41	1997	23
Latvia	2.236	2.21	0.332	1.69	2019	2.81	1998	24
Greece	1.951	1.91	0.215	1.65	2019	2.28	1997	25
France	1.756	1.58	0.431	1.27	2021	2.75	1997	26
Slovenia	1.676	1.52	0.373	1.21	2020	2.38	1997	27
Czechia	1.624	1.57	0.276	1.19	2020	2.13	1997	28
Estonia	1.575	1.42	0.397	1.17	2013	2.46	1998	29
Italy	1.540	1.51	0.348	1.11	2020	2.24	1997	30
San Marino	1.500	1.46	0.279	0.82	2021	2.0	1997	31
Greenland	1.499	1.52	0.184	1.18	2021	1.92	1997	32
Belgium	1.438	1.37	0.364	0.93	2020	2.23	1997	33
Luxembourg	1.342	1.21	0.313	0.93	2020	2.0	1997	34
Denmark	1.189	1.13	0.253	0.88	2021	1.7	1997	35
Malta	1.119	1.07	0.265	0.75	2020	1.58	1997	36
Austria	1.067	0.99	0.200	0.81	2021	1.46	1997	37
Germany	1.052	1.02	0.161	0.83	2021	1.41	1997	38
Cyprus	1.036	1.02	0.185	0.74	2021	1.37	1997	39
Ireland	0.992	0.92	0.215	0.66	2021	1.41	1997	40
Andorra	0.913	0.88	0.157	0.65	2021	1.24	1997	41
Netherlands	0.876	0.81	0.195	0.63	2020	1.23	1997	42
United Kingdom	0.871	0.82	0.116	0.71	2020	1.1	1997	43
Switzerland	0.733	0.69	0.137	0.53	2020	0.98	1997	44
Norway	0.486	0.44	0.098	0.37	2020	0.66	1997	45
Finland	0.463	0.43	0.074	0.38	2013	0.63	1997	46
Iceland	0.463	0.44	0.090	0.36	2021	0.67	1997	47
Sweden	0.345	0.34	0.041	0.27	2021	0.42	2003	48

Monaco ranked third with a mean mortality rate of 3.85 per 100,000, ranging from 4.85 in 1997 to 3.28 in 2020. Serbia and Hungary also ranked high, with mean ASMRs of 3.56 and 3.40, respectively. In Serbia, mortality declined from 4.43 in 1997 to 2.79 in 2021, while in Hungary it decreased from 4.24 in 1999 to 2.71 in 2021. Several Eastern European countries, including Poland (3.26), Bulgaria (3.24), Bosnia and Herzegovina (3.16), and Romania (3.14), also reported relatively high average mortality rates.

On the other end of the spectrum, countries with the lowest mortality rates included Norway, with a mean ASMR of 0.49, ranging from 0.66 in 1997 to 0.37 in 2020. Similarly, Finland and Iceland reported low average rates of 0.46, while Sweden had the lowest mean mortality rate among all countries, at just 0.35 deaths per 100,000. Sweden’s mortality rates remained remarkably stable over time, varying only slightly from 0.42 in 2003 to 0.27 in 2021 (Table 2). Furthermore, Belarus showed the highest standard deviation (SD = 0.82), indicating substantial year-to-year variability, with mortality rates ranging from 1.96 in 2015 to 4.56 in 1998. In contrast, Sweden reported the lowest standard deviation (SD = 0.04), reflecting a stable trend at consistently low mortality levels (Table 2).

**Figure 3:**
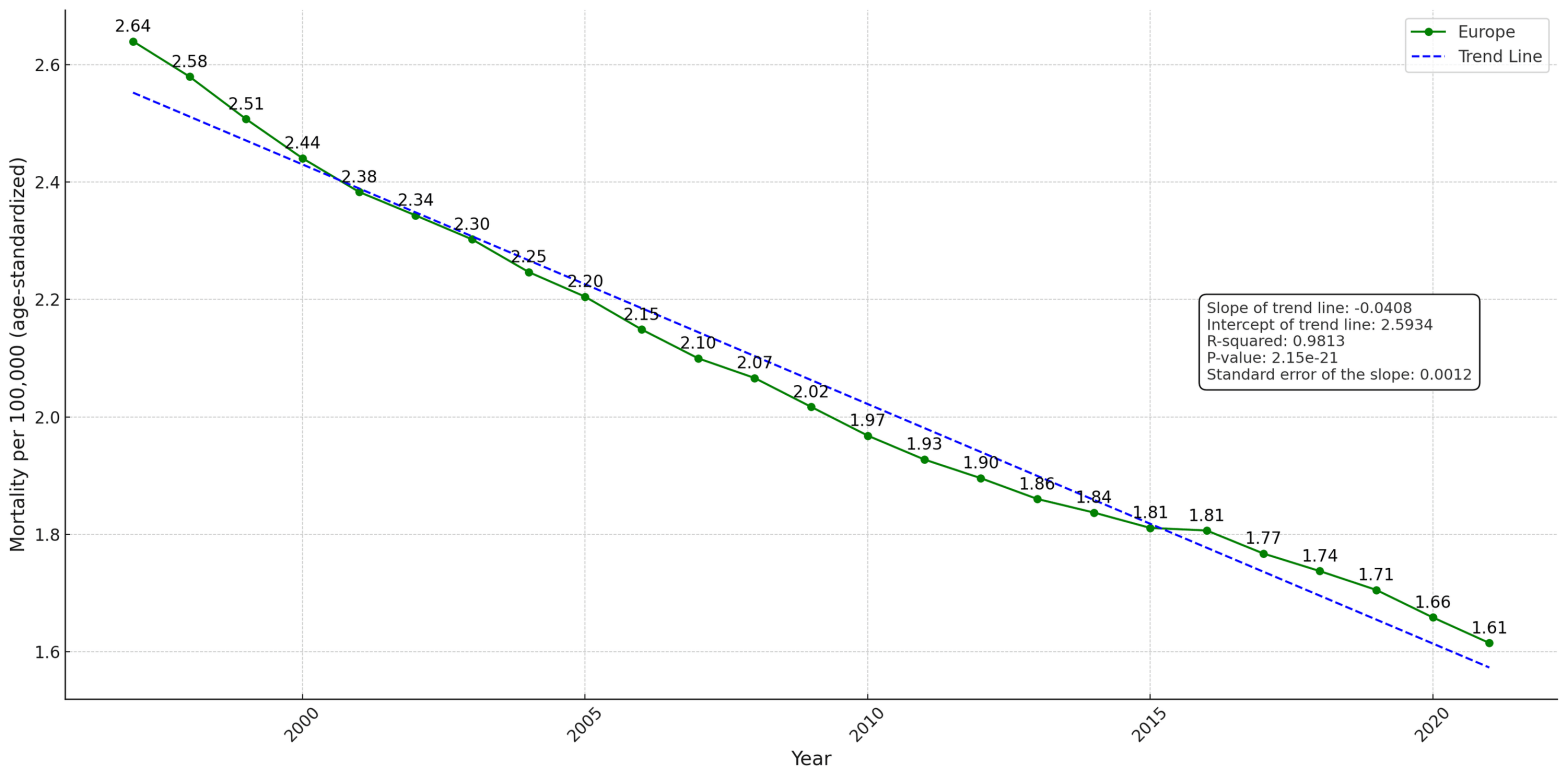
Temporal trend in mean laryngeal cancer mortality per 100,000 population in Europe (age-standardized, 1997–2021) The dashed blue line indicates the linear trend; the green line shows the observed age-standardized mortality rate (ASMR).

Across all European countries, a steady and consistent decline in laryngeal cancer mortality was observed, with the mean ASMR decreasing from 2.64 deaths per 100,000 population in 1997 to 1.61 in 2021 (Figure 3). As indicated by the linear regression analysis, the slope of the trend line was negative (-0.0408), suggesting a gradual annual decrease in mortality over the study period. The R² value was 0.9813 (98%), indicating excellent model fit, while the p-value was <0.001, confirming that the declining trend is statistically significant. Additionally, the standard error of the slope was 0.0012, supporting the precision of the estimate.

**Figure 4:**
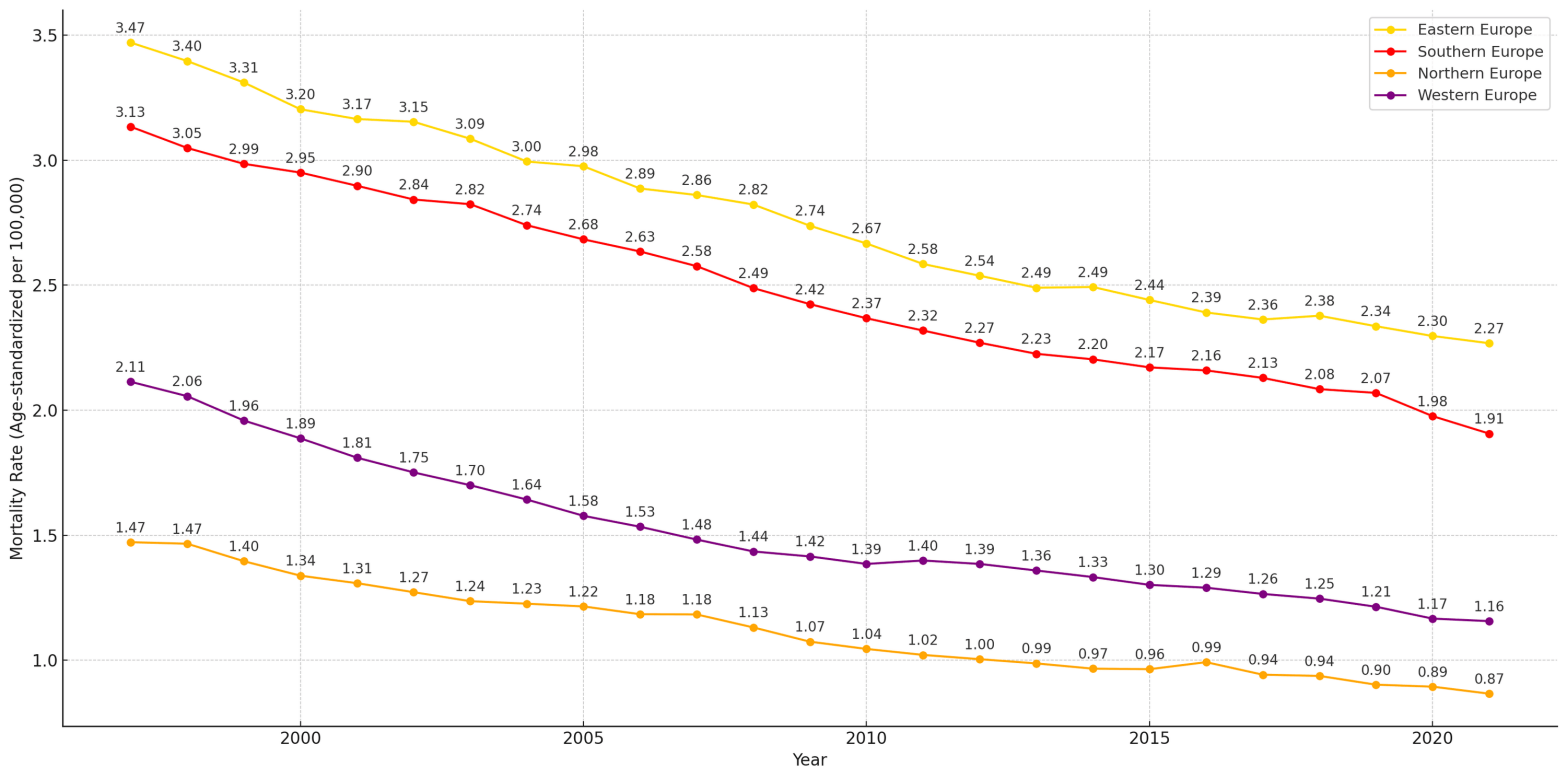
Age-standardized mortality from laryngeal cancer per 100,000 population across European regions, 1997–2021 Regions shown: Eastern Europe (yellow line), Northern Europe (orange line), Southern Europe (red line), and Western Europe (purple line)

At the regional level, ASMRs for laryngeal cancer across the four European subregions from 1997 to 2021 showed notable differences (Figure 4). Eastern Europe recorded the highest mortality rate at the beginning of the study period, with 3.47 deaths per 100,000 population in 1997. A consistent decline followed, reaching 2.27 deaths per 100,000 by 2021. Southern Europe also exhibited elevated mortality early in the period, starting at 3.13 in 1997 and decreasing to 1.91 by 2021. Despite the overall downward trends, both Eastern and Southern Europe maintained the highest mortality rates among the four regions throughout the period.

Western Europe started with an ASMR of 2.11 in 1997 and declined steadily to 1.16 in 2021. Northern Europe recorded the lowest mortality rates throughout the period, decreasing from 1.47 deaths per 100,000 in 1997 to 0.87 in 2021. The overall reduction in Northern Europe was 0.6 deaths per 100,000 population, which was more modest compared to other regions.

Mortality-to-incidence ratio of laryngeal cancer in Europe

**Figure 5:**
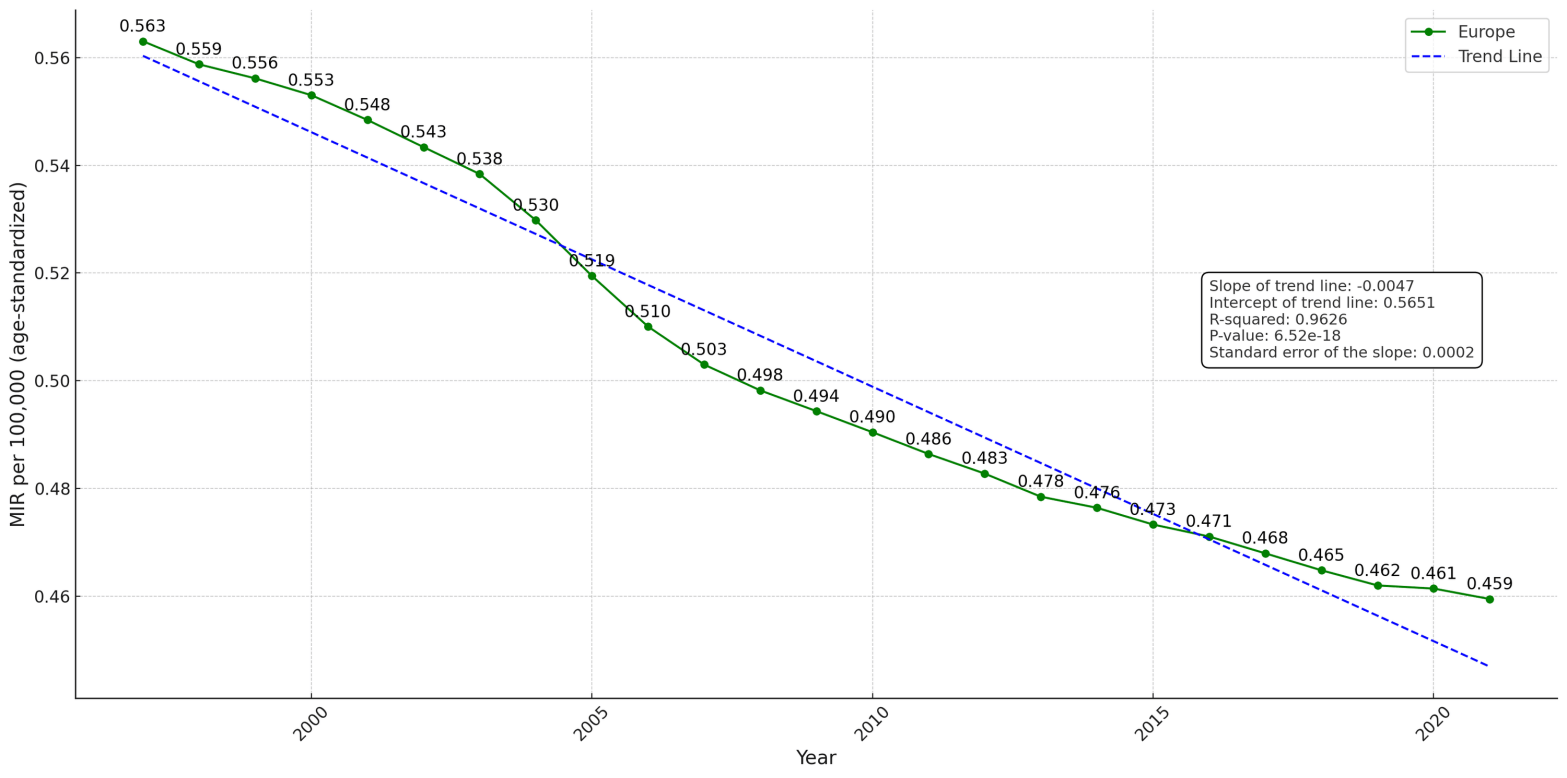
Temporal trend in the average mortality-to-incidence ratio (MIR) for laryngeal cancer per 100,000 population in Europe (age-standardized data, total population, 1997–2021) The dashed blue line represents the linear regression trend; the green line shows the observed MIR values.

The mortality-to-incidence ratio is a key epidemiological indicator in cancer research, reflecting the relationship between the number of deaths and the number of new cases. Figure 5 illustrates the temporal trend of MIRs for laryngeal cancer across Europe between 1997 and 2021. In 1997, the MIR was 0.563, indicating that the age-standardized mortality corresponded to 56.3% of the age-standardized incidence that year, reflecting a relatively elevated mortality burden relative to new diagnoses. Since then, a consistent decline has been observed. By 2007, the MIR had decreased to 0.503, marking a reduction of 0.060 points over the first decade, while by 2021 it had further declined to 0.459, corresponding to a total decrease of 0.104 points over 25 years. Statistical analysis of the trend line revealed a negative slope that was statistically significant (p-value <0.001), with a coefficient of determination (R²) of 0.9626. This suggests that 96.26% of the variation in MIR values over time is explained by the model, confirming a consistent and statistically significant downward trend in MIR across Europe.

To further explore the relationship between mortality and incidence of laryngeal cancer across Europe, we examined the MIR by country and year. Table 3 presents these data, revealing important variations across nations and over time. Countries such as Azerbaijan and Armenia demonstrated persistently high MIRs, starting at 0.857 and 0.803, respectively, in 1997, with only modest declines throughout the study period. These values remained well above the European mean for each corresponding year, indicating a sustained high mortality burden relative to incidence. Similarly, Georgia began with a high MIR of approximately 0.736 in 1997 and, despite minor fluctuations, reported 0.753 in 2021. This stagnation reflects limited improvement in survival outcomes. Montenegro and North Macedonia also reported relatively high MIRs but followed a clearer downward trajectory, suggesting gradual progress over time.

**Table 3:** Temporal evolution of the mortality-to-incidence ratio (MIR) for laryngeal cancer across European countries, 1997–2021

Country	1997	1999	2001	2003	2005	2007	2009	2011	2013	2015	2017	2019	2021
Albania	0.808	0.785	0.768	0.75	0.719	0.689	0.672	0.667	0.661	0.648	0.641	0.632	0.633
Andorra	0.338	0.332	0.329	0.327	0.319	0.309	0.309	0.309	0.301	0.303	0.299	0.296	0.301
Armenia	0.803	0.799	0.793	0.786	0.777	0.765	0.746	0.737	0.729	0.724	0.717	0.697	0.697
Austria	0.468	0.462	0.458	0.456	0.442	0.429	0.427	0.421	0.42	0.42	0.416	0.416	0.42
Azerbaijan	0.857	0.864	0.869	0.87	0.866	0.851	0.838	0.824	0.814	0.802	0.793	0.783	0.782
Belarus	0.705	0.704	0.694	0.684	0.675	0.661	0.641	0.619	0.587	0.56	0.548	0.534	0.528
Belgium	0.395	0.393	0.388	0.386	0.376	0.366	0.358	0.351	0.344	0.34	0.339	0.336	0.332
Bosnia and Herzegovina	0.78	0.743	0.714	0.693	0.676	0.66	0.649	0.64	0.64	0.637	0.628	0.622	0.618
Bulgaria	0.644	0.654	0.639	0.629	0.619	0.61	0.602	0.585	0.574	0.562	0.562	0.56	0.556
Croatia	0.624	0.614	0.601	0.588	0.566	0.55	0.55	0.541	0.526	0.521	0.515	0.5	0.504
Cyprus	0.576	0.556	0.535	0.5	0.444	0.401	0.382	0.379	0.36	0.352	0.35	0.346	0.346
Czechia	0.587	0.573	0.56	0.554	0.531	0.514	0.507	0.498	0.487	0.49	0.484	0.479	0.482
Denmark	0.442	0.44	0.44	0.426	0.402	0.377	0.368	0.364	0.355	0.348	0.349	0.344	0.346
Estonia	0.542	0.535	0.515	0.499	0.457	0.421	0.405	0.388	0.371	0.363	0.359	0.359	0.355
Finland	0.321	0.314	0.314	0.307	0.284	0.269	0.267	0.268	0.264	0.258	0.262	0.259	0.252
France	0.38	0.37	0.368	0.353	0.324	0.299	0.29	0.279	0.269	0.265	0.266	0.263	0.263
Georgia	0.736	0.747	0.747	0.743	0.751	0.765	0.771	0.763	0.765	0.765	0.754	0.755	0.753
Germany	0.418	0.414	0.405	0.405	0.396	0.385	0.382	0.383	0.384	0.381	0.379	0.375	0.372
Greece	0.404	0.398	0.397	0.394	0.385	0.373	0.367	0.357	0.367	0.375	0.371	0.369	0.368
Greenland	0.741	0.733	0.731	0.72	0.711	0.698	0.69	0.673	0.664	0.657	0.645	0.639	0.631
Hungary	0.627	0.607	0.588	0.575	0.567	0.559	0.549	0.542	0.543	0.542	0.533	0.519	0.511
Iceland	0.335	0.333	0.327	0.323	0.314	0.3	0.299	0.299	0.299	0.296	0.291	0.29	0.283
Ireland	0.456	0.447	0.43	0.418	0.378	0.355	0.342	0.332	0.324	0.322	0.315	0.308	0.274
Italy	0.416	0.412	0.414	0.405	0.385	0.374	0.375	0.369	0.37	0.368	0.369	0.364	0.368
Latvia	0.71	0.712	0.704	0.686	0.68	0.67	0.658	0.655	0.64	0.633	0.618	0.604	0.595
Lithuania	0.707	0.696	0.675	0.67	0.669	0.678	0.665	0.655	0.648	0.65	0.64	0.622	0.613
Luxembourg	0.407	0.403	0.405	0.396	0.368	0.349	0.341	0.34	0.336	0.331	0.33	0.326	0.325
Malta	0.461	0.448	0.444	0.435	0.42	0.404	0.392	0.382	0.37	0.358	0.349	0.34	0.338
Monaco	0.356	0.355	0.357	0.355	0.344	0.334	0.33	0.327	0.322	0.32	0.318	0.315	0.314
Montenegro	0.641	0.636	0.626	0.613	0.597	0.579	0.571	0.563	0.553	0.546	0.54	0.53	0.536
Netherlands	0.347	0.35	0.346	0.34	0.315	0.298	0.295	0.294	0.291	0.289	0.291	0.292	0.294
North Macedonia	0.784	0.769	0.754	0.732	0.709	0.689	0.671	0.661	0.657	0.649	0.646	0.65	0.64
Norway	0.314	0.312	0.311	0.299	0.282	0.268	0.264	0.262	0.255	0.258	0.265	0.273	0.278
Poland	0.743	0.719	0.704	0.695	0.686	0.679	0.674	0.662	0.648	0.647	0.644	0.641	0.638
Portugal	0.771	0.765	0.753	0.739	0.71	0.684	0.677	0.664	0.65	0.652	0.646	0.643	0.643
Republic of Moldova	0.77	0.768	0.768	0.761	0.752	0.745	0.732	0.715	0.697	0.681	0.661	0.642	0.639
Romania	0.698	0.682	0.666	0.646	0.622	0.594	0.579	0.566	0.55	0.54	0.531	0.525	0.524
Russian Federation	0.756	0.76	0.761	0.754	0.718	0.695	0.676	0.659	0.637	0.617	0.601	0.583	0.58
San Marino	0.338	0.335	0.335	0.335	0.329	0.323	0.321	0.321	0.322	0.322	0.322	0.321	0.311
Serbia	0.671	0.66	0.648	0.631	0.597	0.571	0.556	0.544	0.534	0.523	0.515	0.507	0.503
Slovakia	0.68	0.673	0.66	0.651	0.64	0.628	0.622	0.609	0.598	0.587	0.579	0.566	0.567
Slovenia	0.565	0.555	0.537	0.523	0.48	0.442	0.436	0.428	0.41	0.395	0.386	0.382	0.385
Spain	0.443	0.437	0.436	0.423	0.397	0.379	0.377	0.374	0.365	0.366	0.36	0.354	0.355
Sweden	0.307	0.303	0.311	0.302	0.289	0.272	0.267	0.272	0.265	0.265	0.274	0.272	0.276
Switzerland	0.329	0.321	0.316	0.317	0.302	0.293	0.291	0.291	0.289	0.288	0.29	0.289	0.29
Turkey	0.786	0.768	0.745	0.722	0.683	0.645	0.623	0.601	0.593	0.583	0.563	0.548	0.535
Ukraine	0.692	0.701	0.705	0.7	0.675	0.65	0.636	0.631	0.637	0.639	0.627	0.625	0.622
United Kingdom	0.348	0.339	0.334	0.327	0.307	0.295	0.289	0.283	0.281	0.28	0.28	0.28	0.278

In contrast, Ireland and Norway maintained some of the lowest MIRs in Europe, with the MIR for Ireland decreasing from 0.456 in 1997 to 0.274 in 2021. Greece demonstrated a decline, from 0.404 in 1997 to 0.368 in 2021, aligning with the general European trend. Other Western and Northern European countries, including the United Kingdom, Sweden, and Luxembourg, also reported lower MIRs, characterized by minor fluctuations and overall stability. Finland recorded one of the lowest MIRs in Europe, decreasing from 0.321 in 1997 to 0.252 in 2021, while France followed a similar pattern, with MIR values falling from 0.380 to 0.263. These consistently low ratios confirm the favorable cancer survival profiles achieved in these countries.

**Figure 6:**
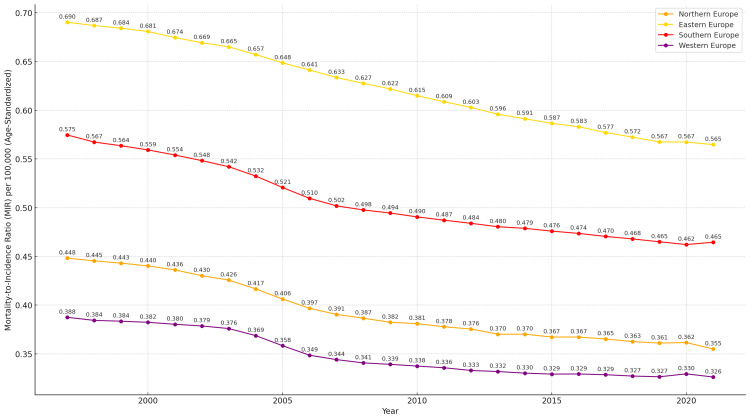
Temporal trends in the average mortality-to-incidence ratio (MIR) per 100,000 population for laryngeal cancer across European subregions (age-standardized, both sexes, 1997–2021) Regions represented: Northern Europe (orange line), Eastern Europe (yellow line), Southern Europe (red line), and Western Europe (purple line). The lines illustrate the observed annual MIRs from 1997 to 2021 across European subregions.

At the regional level (Figure 6), all European subregions exhibited a declining trend in the MIR for laryngeal cancer between 1997 and 2021. However, notable differences emerged in both the average MIR levels and the pace of reduction across regions. Eastern Europe consistently reported the highest MIR throughout the period, beginning at 0.690 in 1997 and gradually decreasing to 0.565 in 2021. Southern Europe also exhibited high MIR values, starting at 0.575 in 1997 and declining to 0.465 by 2021. Although Southern Europe demonstrated a significant overall decrease, it maintained particularly elevated MIR levels in the earlier years of the analysis.

Northern Europe started at an intermediate level, with an MIR of 0.448 in 1997, and declined steadily to 0.355 by 2021. While its MIR remained lower than that of Eastern and Southern Europe, Northern Europe still showed potential for further improvement when compared to Western Europe.

Western Europe recorded the lowest MIR values over the entire study period, beginning at 0.388 in 1997 and reaching the lowest regional MIR of 0.326 in 2021. These values reflect the most favorable mortality-to-incidence ratio among the European subregions, indicating better survival outcomes relative to new cases, both compared to other regions and to the overall European average.

## Discussion

The incidence of laryngeal cancer in Europe represents a significant public health concern, reflecting both regional disparities and long-term trends that influence the development of prevention and intervention strategies. Our findings provide a comprehensive overview of the disease’s evolution over the past 25 years (1997-2021), highlighting variations between geographic regions.

The overall downward trend in the age-standardized incidence of laryngeal cancer across Europe during the study period is noteworthy. The mean incidence decreased from 4.64 to 3.40 cases per 100,000 population, painting a positive picture of the effectiveness of public health efforts aimed at reducing tobacco and alcohol consumption, the implementation of anti-smoking campaigns, and improvements in occupational safety conditions [11-19]. Increasing efforts across European countries to limit exposure to tobacco smoke, both in indoor and outdoor public spaces, have likely contributed to the reduction in passive smoking and, by extension, to the decline in laryngeal cancer cases associated with such exposure [11,20-21].

At the regional level, the highest incidence rates of laryngeal cancer throughout the study period were observed in Southern, Eastern, and Western Europe. These findings may be attributed to historically elevated levels of tobacco use and alcohol consumption, deeply embedded in the lifestyle and culture of certain populations in these regions, as well as to broader socioeconomic and cultural determinants [22-27]. In addition to overall prevalence, variations in tobacco type, alcohol consumption patterns (heavy or binge drinking), and cumulative duration of exposure (years of smoking or alcohol use) have been highlighted as important contributors. Differences in socioeconomic status and prevention policy implementation may also have limited the effectiveness of public health measures in some countries, thereby sustaining higher incidence levels [26-30].

In particular, although Southern Europe demonstrated a declining trend, incidence rates remained higher than those in other European subregions. This may reflect insufficient implementation of prevention policies, such as limited awareness of risk factors and challenges in adopting preventive health behaviors [28-30]. In contrast, Northern Europe recorded the lowest incidence rates and the smallest variation over time, reflecting the effectiveness of comprehensive public health measures, including strict tobacco control policies, improved occupational safety standards, and greater adoption of health-conscious lifestyles [11-12,18,24-25,31].

At the country level, the highest average incidence rates were reported in Monaco (11.49 per 100,000), Montenegro (9.31), and Serbia (6.19). The elevated burden in these countries is strongly associated with cultural and behavioral determinants, particularly high smoking prevalence and alcohol consumption, which remain the major established risk factors for laryngeal cancer [3-4,32-35]. Additional contributors, such as occupational exposures and environmental pollution, may also play a role in shaping these trends [3-4]. On the other end of the spectrum, the lowest incidence rates were observed in Sweden (1.22), Iceland (1.50), and Finland (1.65), likely reflecting the successful implementation of public health policies to reduce tobacco and alcohol use, as well as differences in dietary patterns and lower levels of air pollution [22-23,36-38].

Mortality, as a critical indicator for understanding the severity and prognosis of laryngeal cancer [4,39], revealed significant temporal trends and geographical differences throughout our analysis, further highlighting the multifactorial nature of the disease. More specifically, between 1997 and 2021, the age-standardized mortality rate for laryngeal cancer in Europe followed a consistently downward trajectory, declining from 2.64 deaths per 100,000 population in 1997 to 1.61 in 2021. This trend was statistically significant (p < 0.001). The sustained reduction in mortality is likely associated with increased public awareness regarding early diagnosis, advancements in therapeutic interventions, broader access to specialized healthcare services, and, more generally, the strengthening of oncological care, particularly in Western and Northern European countries [36,38,40-42].

Beyond the overall reduction in laryngeal cancer mortality across Europe, geographical disparities between subregions are equally noteworthy. Countries in Eastern and Southern Europe consistently reported the highest mortality rates throughout the study period. Specifically, in Eastern Europe, mortality declined from 3.47 deaths per 100,000 population in 1997 to 2.27 in 2021, while in Southern Europe, the corresponding rates were 3.13 and 1.91, respectively.

The persistently elevated mortality in these regions may be linked to limited access to modern therapeutic approaches, especially during the early years of the study period, as well as to broader health system inequalities that hinder timely diagnosis and treatment efficacy [43-45]. In addition, low availability of specialized oncology centers and the absence of comprehensive screening programs may have contributed to delayed diagnoses, with many cases detected at advanced stages, which are associated with poorer survival outcomes [45-47]. In contrast, Western and Northern Europe reported the lowest mortality rates for laryngeal cancer. Western Europe saw a decline from 2.11 deaths per 100,000 in 1997 to 1.16 in 2021, while Northern Europe recorded even lower levels, 1.47 in 1997 and 0.87 in 2021. These favorable outcomes reflect higher investments in healthcare services, enhanced quality of medical care, broader access to specialized oncology facilities, and the use of more advanced therapeutic protocols [36-38,40-42,48].

At the country level, the highest laryngeal cancer mortality rates were observed in Montenegro and North Macedonia, with values ranging from 6.07 to 3.2 deaths per 100,000 population over the study period. Both countries have historically exhibited high levels of tobacco and alcohol consumption, which are the well-established risk factors linked to poorer prognosis, increased treatment complications, and higher mortality rates in head and neck cancer patients [34,49-54]. On the other hand, Sweden and Iceland consistently maintained the lowest mortality rates, remaining below 0.5 deaths per 100,000 population. This favorable trend may be attributed to increased investment in public health, widespread access to high-quality healthcare services, and substantial funding for oncology research, all of which have likely contributed to more effective treatment strategies and improved outcomes [37-38].

Analysis of incidence and mortality data revealed substantial disparities among European countries, highlighting both a general decline in laryngeal cancer cases and improvements in patient survival. However, the mortality-to-incidence ratio serves as a critical indicator, offering insights into the relationship between new cases and cancer-related deaths, and enabling a more comprehensive assessment of overall prognosis [8-9]. Our findings demonstrate a clear and significant downward trend in MIRs across Europe, indicating remarkable progress in the management of laryngeal cancer. The MIR steadily declined from 0.563 in 1997 to 0.459 in 2021, the lowest value observed during the study period. This trend was statistically significant (p < 0.001) and demonstrated excellent fit in the linear regression model (R² = 96.26%).

Despite these encouraging developments, notable geographic disparities persist. Countries in Eastern Europe continue to face significant challenges, reflected in persistently higher MIR values. In contrast, nations in Northern and Western Europe have achieved substantially lower MIRs, suggesting better access to early diagnosis, advanced therapeutic interventions, and more equitable cancer care systems [36-38,41-42,48]. More specifically, Eastern Europe consistently recorded the highest MIR values for laryngeal cancer throughout the study period, starting at 0.690 in 1997 and decreasing to 0.565 by 2021. Countries within this region, such as Azerbaijan and Armenia, maintained some of the highest MIRs among all European nations. For instance, Azerbaijan reported an MIR of 0.857 in 1997 and, despite a slight decline, still exhibited a high MIR of 0.782 in 2021. Georgia showed an initial improvement, but the MIR subsequently increased slightly from 0.736 in 1997 to 0.753 in 2021, highlighting persistent challenges in improving survival outcomes.

These elevated MIR values likely reflect a confluence of factors related to healthcare system performance, public health policies, and population behavior. Persistently high rates of tobacco and alcohol consumption in all three countries contribute significantly to poor prognoses. Furthermore, limited access to diagnostic and therapeutic services, particularly in rural and remote areas, unequal distribution of healthcare resources, and underfunding of public health infrastructure remain major obstacles to effective cancer management. The absence of comprehensive national cancer control plans and early detection programs further restricts timely diagnosis and intervention, thereby exacerbating disease outcomes [55-57].

Similarly, Southern Europe, despite demonstrating substantial improvements, began with a relatively high MIR of 0.575 in 1997, which declined to 0.465 by 2021. These elevated MIR levels may be attributed to several factors, including the limited implementation of structured prevention programs, delayed diagnoses, and inequalities in both access to and quality of treatment. While progress has been made, structural barriers to comprehensive oncology care persist in certain Southern European countries [49-50,58-60].

Western Europe consistently recorded the lowest MIR values throughout the study period, beginning at 0.388 in 1997 and reaching 0.326 in 2021. This favorable profile likely reflects more effective management of laryngeal cancer cases, supported by improved access to diagnostic services and advanced therapeutic interventions. Countries such as France, Germany, Austria, and Luxembourg have invested heavily in oncology through the expansion of cancer networks, adoption of precision technologies, and establishment of multidisciplinary approaches. These countries have also minimized access barriers via strong public funding and low out-of-pocket costs for patients [41-42,48,61-62].

Northern Europe, although showing slightly higher MIR levels compared to Western Europe, followed a steady downward trajectory, reaching 0.355 by 2021. Countries such as the United Kingdom, Finland, Sweden, and Iceland reported some of the lowest MIR values in Europe, with consistent year-on-year reductions. For example, Finland’s MIR declined from 0.321 in 1997 to 0.252 in 2021, and that for Sweden from 0.307 to 0.276 over the same period. These low MIR values reflect the successful implementation of screening programs, strengthening of primary care, and widespread access to high-quality healthcare services that support timely diagnosis and optimal treatment [36-38,63]. Moreover, increased public awareness through extensive education and prevention campaigns, such as those led by Cancer Research UK, has promoted early health-seeking behaviors and contributed to improved prognosis. These advances are further supported by the European strategy Europe’s Beating Cancer Plan, which, through the EU4Health program, aims to reduce inequalities, strengthen prevention, and ensure equitable access to high-quality care for all EU citizens [36-38,63-65].

These regionally targeted efforts provide a valuable roadmap for scaling up evidence-based strategies in laryngeal cancer control across Europe, highlighting the importance of equity, early detection, and robust oncology care infrastructure.

Strengths and limitations

A major strength of this study lies in its comprehensive and longitudinal approach, analyzing age-standardized incidence, mortality, and mortality-to-incidence ratios for laryngeal cancer across all European countries over a 25-year period (1997-2021). By utilizing publicly available, high-quality data from the Global Burden of Disease database, the study ensured methodological consistency and comparability across countries and regions. The stratification of results by European subregions provided additional insights into geographic disparities, enabling a better understanding of epidemiological trends and public health implications at both national and regional levels. Furthermore, the inclusion of MIR offered a more nuanced assessment of prognosis and health system performance beyond incidence or mortality alone.

However, this study also has limitations. First, the use of aggregated national-level data may obscure within-country disparities, particularly between urban and rural areas or between socioeconomic groups. Moreover, the accuracy and completeness of GBD data may vary between countries, depending on the strength of national cancer registries and reporting systems, which could introduce bias in cross-country comparisons. Second, due to its ecological design, this study could not assess causal relationships or account for individual-level risk factors such as tobacco or alcohol use, or occupational exposures. Third, while MIR is a useful proxy for cancer survival, it does not replace detailed survival analysis based on individual-level data. Finally, the choice of Microsoft Excel for statistical analysis could be considered a limitation, as it does not provide the advanced functions of specialized statistical software. However, for the descriptive and regression analyses performed here, which required only basic statistical procedures, Excel was deemed appropriate and sufficient.

## Conclusions

The 25-year analysis demonstrated a significant and steady decline in the incidence, mortality, and mortality-to-incidence ratio of laryngeal cancer across Europe, yet striking regional disparities remain. Northern Europe achieved the lowest incidence and mortality rates, while Western Europe, despite relatively higher incidence, maintained the lowest MIR overall, reflecting advanced treatment capabilities and improved survival. Countries such as Finland, France, Ireland, Sweden, and Norway consistently reported the most favorable MIRs, underscoring the impact of robust prevention, early detection, and well-resourced healthcare systems.

In contrast, Eastern and Southern Europe continue to bear the highest burden, highlighting the need for urgent, region-specific action. Sustainable public health strategies, centered on risk factor reduction, timely diagnosis, equitable treatment access, and strengthened oncology infrastructure, are essential to closing the survival gap. Without decisive policy interventions, these disparities will persist, undermining the goal of reducing the overall cancer burden across Europe.
